# Schmidt on Venereal Diseases

**Published:** 1903-01

**Authors:** 


Schmidt on Venereal Diseases.—Lea's Series of Medical Epitomes. A manual of genito-urinary and venereal diseases for the use of students and practitioners. By Louis E. Schmidt, M. D., of the Chicago Polyclinic. In one handy 12mo. volume of 250 pages, with 21 illustrations. Cloth, $1.00, net. Lea Brothers & Co., Publishers, Philadelphia and New York, 1902.

In this, the first of Lea's Series of Medical Epitomes, the author has prepared a brief but comprehensive and well written manual on genito-urinary and venereal disease. In order to avoid breaking the continuity of the text, a series of questions intended for the use of students are placed at the end of each chapter.

The work is rather too much condensed to be of value to practitioners, it may be recommended as a safe and very valuable aid for undergraduate students. R.

